# Effects of Continuous Nitrogen Fertilizer Application on the Diversity and Composition of Rhizosphere Soil Bacteria

**DOI:** 10.3389/fmicb.2020.01948

**Published:** 2020-08-21

**Authors:** Ning Ren, Yang Wang, Youliang Ye, Yanan Zhao, Yufang Huang, Wen Fu, Xv Chu

**Affiliations:** Agricultural Green Development Engineering Technology Research Center, College of Resources and Environment, Henan Agricultural University, Zhengzhou, China

**Keywords:** soil bacterial, average relative richness, soil pH, long-term N application, wheat yield

## Abstract

Little has been reported on the effects of long-term fertilization on rhizosphere soil microbial diversity. Here, we investigated the effects of long-term continuous nitrogen (N) fertilization on the diversity and composition of soil bacteria using data from a 10-year field experiment with five N application rates (0, 120, 180, 240, and 360 kg N hm^–2^). The results revealed varying degrees of reduction in the numbers of bacterial operational taxonomic units (OTUs) in response to the different N application rates. The highest wheat yield and number of proprietary bacterial OTUs were found in the N input of 180 kg N hm^–2^. In terms of average relative richness, the top seven phyla of soil bacteria in the rhizosphere of wheat after long-term nitrogen application were Proteobacteria, *Actinobacteria, Acidobacteria, Chloroflexi, Bacteroidetes, Gemmatimonadetes, and Patescibacteria*. Among these, *Proteobacteria* and *Gemmatimonadetes* were found to be unaffected by the nitrogen fertilizer and soil environmental factors (pH, C/N ratio, and NO_3_^–^ concentration), whereas *Acidobacteria* and *Actinobacteria* showed significant positive and negative correlations, respectively, with soil pH. The richness of *Actinobacteria* significantly increased in the N_180_ treatment. *Patescibacteria* and *Bacteroidetes* showed significant positive correlations with soil NO_3_^–^ and wheat yield, and the average relative richness of these two phyla was high under long-term application of the N_180_ treatment. These findings indicate that the relative richness of *Patescibacteria* and *Bacteroidetes* can affect wheat yield. In conclusion, the results of our 10-year field experiments clearly show that long-term N fertilization can significantly affect most of the dominant soil bacterial species *via* changing the soil pH. The richness of *Actinobacteria* can serve as an indicator of a decreased soil pH caused by long-term N fertilization.

## Introduction

Wheat is an essential commodity grain and strategic grain reserve in China. It plays an extremely important role in ensuring national food security (Hu et al., 2016). The arable land in China accounts for approximately 9% of the world’s total, and the crops produced on this land, of which wheat production contributes 20.30%, feed some 22% of the world’s population (Ma et al., 2013). Nitrogen (N) fertilizer, as the basis of the sustained high grain yields, plays a decisive role in agricultural production (Islam et al., 2015), with the appropriate application of N fertilizer contributing to increases in the absorption area and the activity of crop root systems and promotion of the emergence of productive tillers.

An optimized N management strategy (application of 128 kg N hm^–2^) has been shown to be significantly lower than the amount typically applied by farmers (325 kg N hm^–2^) without any appreciable loss in wheat grain yield (Cui et al., 2008). However, as the beneficial effects of using N fertilizer in the field are very obvious, most farmers preferentially apply excessive amounts with the aim of obtaining higher yields. China’s N fertilizer consumption has grown by 121.02% from 1980 to 2018 (National bureau of statistics, 2019). Low N fertilizer inputs can lead to a reduction in soil fertility, which limits the availability of adequate nutrients during crop growth and development, resulting in crop yield losses. However, high N inputs may also result in lower yields and tend to be conducive to the emergence and frequency of pests and diseases and, moreover, can have detrimental environmental effects, such as water eutrophication (Tesoriero et al., 2009), increased greenhouse gas emissions (Luo et al., 2016), and increases in soil acidity and salinity (Han et al., 2017). Thus, appropriate N fertilizer inputs are the key to balancing high crop yields and environmental compatibility.

The activities of the soil microorganisms can directly enrich the physical, chemical, and biological properties of soils and have significant positive effects on the utilization of rhizosphere nutrient resources by plants (Gasparatos et al., 2011). Bacteria are the most abundant and diverse group of soil microorganisms (Jason et al., 2005), and they play vital roles in the decomposition of organic matter, transformation of nutrients, degradation of pollutants, maintenance of ecosystem sustainability, and regulation of soil productivity for plant growth (Koranda et al., 2013; Oburger et al., 2016).

In turn, the soil ecosystem, *via* diverse environmental factors such as illumination intensity as well as the physical and chemical properties, plays a prominent role in influencing the composition and activity of soil bacterial communities (Lagomarsino et al., 2007). Bacterial diversity, which includes the genetic variability within species and the number and relative richness of the taxa and functional traits in communities, plays a vital role in ecosystem functionality. Rhizosphere soil bacterial diversity is particularly sensitive to environmental changes, such as those initiated by anthropogenic disturbances, including long-term nutrient inputs (Ai et al., 2013), and long-term N-free treatments have revealed a trend toward higher richness and diversity (Eo and Park, 2016).

The rhizosphere microorganisms have an important influence on the growth and development of plants, whereas plants have the ability to change the soil environment by secreting bioactive molecules into the rhizosphere to regulate the local growth conditions (Chen et al., 2019). Changes in the composition of root-related microbiota during plant development are caused by changes in root exudates (Lennon and Jones, 2011). It was pointed out that the composition of the wheat rhizosphere bacteria community was closely related to the organic carbon released by the roots. In addition, rhizosphere bacteria were the driving factors for the secretion of organic acids and other substances by the roots of wheat and played an important role in the uptake and transformation of nutrients in the roots of wheat (Haichar et al., 2008).

Long-term fertilizer inputs inevitably have the effect of altering the soil pH, which is considered to be an important determinant of bacterial diversity and community structure and also modifies the effects of fertilizer nitrification. For example, the increased nitrification of soil promoted by the application of chemical N fertilizers is related to changes in the community richness and the structure of ammonia-oxidizing bacteria (Chen et al., 2014). Inorganic fertilizer in the soil is transformed into nutrients that can be directly absorbed by crop roots through the metabolic activities of soil microorganisms. However, excessive fertilization will ultimately affect the richness of nitrifying bacteria in the soil (Geisseler and Scow, 2014).

Previous studies have indicated that fertilization changes the diversity, community structure, and activity of soil microorganisms (Wei et al., 2018; Chen et al., 2019). The long-term application of chemical fertilizers can significantly alter the structure and diversity of bacterial communities (Ge et al., 2008), and numerous recent studies have used sequencing technology to examine the effects of fertilization on the diversity and structural composition of soil microbial communities (Mchugh and Schwartz, 2015; Sun et al., 2015). However, most of these studies have tended to focus on the relationship between the overall change in bacterial communities and the nutrient uptake of crops, as well as the relationship between soil bacteria and the ecological environment (Ortiz-Castro et al., 2009; Maron et al., 2011; Jansson and Hofmockel, 2019). In contrast, there has been relatively little research conducted on the specific functional groups of bacteria affected by N fertilization or on the relationships between wheat yield trend and soil bacterial dynamics under the long-term application of N fertilizer.

In this study, we established five fertilization treatments, in which different amounts of N fertilizer were applied to the soil over 10 consecutive years. Rhizosphere soil bacterial populations were analyzed using high-throughput sequencing of 16S ribosomal RNA (rRNA) gene amplicons, and we examined trends in the diversity and richness of wheat rhizosphere soil bacterial communities relating to the different N fertilizer levels and assessed their correlations with wheat production. Furthermore, we elucidated the mechanisms underlying the optimization of bacterial richness and the relationship between wheat yield and rhizosphere soil bacteria and also discussed the relationship between functional bacteria and the soil environment. We believe that the findings of this study provide a valuable theoretical basis that will contribute to guiding fertilization practices.

## Materials and Methods

### Field Experiment and Sample Collection

The experiment was carried out in Yuzhou City, Henan Province, China (34°27′ N, 113°34′ E), with an average annual temperature of 13–16°C and an average annual precipitation of 650 mm. The soil of the study area was fluvoaquic at pH 7.54, soil organic matter (SOM) of 20.5 g kg^–1^, and total nitrogen (TN) of 0.92 g kg^–1^. The proposed investigation was arranged in a randomized complete block with three replications, under five N treatments: N_0_ (0 kg N hm^–2^), N_120_ (120 kg N hm^–2^), N_180_ (180 kg N hm^–2^), N_240_ (240 kg N hm^–2^), and N_360_ (360 kg N hm^–2^). N (urea) was applied at two intervals. Between them, 50% N was used as the base fertilizer and 50% N was topdressing at jointing. Phosphate fertilizer (90 kg P_2_O_5_ hm^–2^), in the form of calcium superphosphate, and potassium (90 kg K_2_O hm^–2^), employing potassium chloride, were applied as the basal dose. The base fertilizer (urea) was manured manually before the plowing, and the topdressing (urea) was applied by artificial band.

The study was commenced in 2009, and subsequently, wheat rhizosphere soil was collected during the first, fifth, and 10th harvest years of the field experiment at wheat harvest stage. The rhizosphere soil samples were used with a root drill, and 10 wheat rhizosphere soil samples were taken from each plot. The rhizosphere samples in this study were strictly defined as soil within 2 mm of the root surface (DeAngelis et al., 2009). The roots were gently shaken to remove any loose clumps of soil, after which the rhizosphere samples were collected by brushing off the remaining soil deliberately.

### Determination of TN, SOM, pH, NO_3_^–^, and Yield

Rhizosphere soil was air-dried and sieved at 0.149 mm to prepare the soil samples for the following analyses. For the total N (TN) determination, the soil samples were boiled with concentrated hydrogen peroxide of sulfuric acid and determined by adopting the Kjeldahl method (Bremner and Mulvaney, 1982). The Automatic Kjeldahl apparatus (K9840, Hanon Instruments Co., Ltd., China) was utilized in the test process. The soil organic carbon (SOC) concentration was determined by the classical potassium dichromate oxidation–ferrous sulfate titration method (Shaw, 1959). The Numerical Show Constant Temperature Oil-bathing (HH-WO-2L-50L, Shanghai Xuhang Scientific Instrument Co. Ltd., China) is used in the oxidation process. For TN and SOC, 0.5 g soil sample was used. Determination of soil nitrate (NO_3_^–^) was done by the KCL leaching method (Page et al., 1982). For soil NO_3_^–^, 0.5 g fresh soil sample was weighed, extracted with 0.01 mol L^–1^ KCl solution, and analyzed using an automated flow analysis instrument (AA3, Seal, Germany). The average yield of each plot was calculated by taking three wheat plots of 1 m^2^ for each plot and then the wheat yield of the different treatments was calculated.

### DNA Extraction and PCR Amplification

Microbial DNA was extracted from rhizosphere soil samples using the FastDNA® SPIN Kit for Soil according to the manufacturer’s protocols. The FastDNA® SPIN kit consists of three parts: (i) cracking; (ii) homogenizing reagent (reagents can extract genomic DNA with minimal RNA contamination); and (iii) DNA purification and elution reagents (program purified DNA with a special silica gel matrix while eliminating the contaminants and inhibiting concurrent reactions). The final DNA concentration and purification were determined by a NanoDrop 2000 UV–Vis spectrophotometer (Thermo Fisher Scientific, Wilmington, NC, United States), and DNA quality was checked by 1% agarose gel electrophoresis. The V3–V4 hypervariable regions of the bacteria 16S rRNA gene were amplified with primers 338F (5′-ACTCCTACGGGAGGCAGCAG-3′) and 806R (5′-GGACTACHVGGGTWTCTAAT-3′) (Mori et al., 2014; Nan et al., 2016) by a thermocycler PCR system (GeneAmp 9700, ABI, United States). The PCR reactions were conducted using the following program: 3 min of denaturation at 95°C, 27 cycles of 30 s at 95°C, 30 s for annealing at 55°C, and 45 s for elongation at 72°C, and a final extension at 72°C for 10 min. PCR reactions were performed in triplicate 20 μl mixture containing 4 μl of 5 × FastPfu buffer, 2 μl of 2.5 mM dNTPs, 0.8 μl of each primer (5 μM), 0.4 μl of FastPfu polymerase, and 10 ng of template DNA. The resulting PCR products were extracted from a 2% agarose gel and further purified using the AxyPrep DNA Gel Extraction Kit (Axygen Biosciences, Union City, CA, United States) and quantified using QuantiFluor^TM^-ST (Promega, United States) according to the manufacturer’s protocol.

### Illumina MiSeq Sequencing

Purified amplicons were pooled in equimolar concentrations and paired-end sequenced (2 × 300) on an Illumina MiSeq platform (Illumina, San Diego, United States) according to the standard protocols by Majorbio Bio-Pharm Technology Co., Ltd. (Shanghai, China). The raw reads were deposited into the NCBI sequence read archive (SRA) under the submission ID SUB7174545. The project can also be accessed in NCBI under BioProject ID 613763 (accession PRJNA613763).

### Processing of Sequencing Data

Raw fastq files were demultiplexed, quality-filtered by Trimmomatic, and merged by FLASH with the following criteria: (i) The reads were truncated at any site receiving an average quality score <20 over a 50 bp sliding window. (ii) Primers were exactly matched, allowing two nucleotides mismatching, and reads containing ambiguous bases were removed. (iii) Sequences with overlaps longer than 10 bp were merged according to their overlap sequence.

Operational taxonomic units (OTUs) were clustered with a 97% similarity cutoff using UPARSE (version 7.1),^1^ and chimeric sequences were identified and removed using UCHIME. The taxonomy of each 16S rRNA gene sequence was analyzed by the RDP Classifier algorithm^2^ against the Silva (SSU123) 16S rRNA database using a confidence threshold of 70%.

### Statistical Analyses

Chao richness and Shannon diversity were generated using QIIME (Quantitative Insights Into Microbial Ecology) 1.9.1 (Caporaso et al., 2010). To determine the impact of environmental factors (soil C/N ratio, soil pH, and NO_3_^–^) on the microbial community structure, we performed variance partitioning of the Bray–Curtis distances of all samples of rhizosphere soil with distance-based redundancy analysis (db-RDA). Circos, conducted using Circos-0.67-7,^3^ was adopted to assess the correspondence between the samples and species. Significance was determined by one-way analysis of variance (ANOVA) with *post hoc* contrasts by the Student–Newman–Keuls test. The statistical significance for all tests was set at *P* < 0.05. Heat maps were constructed using R v.2.15.2 (R Foundation for Statistical Computing)^4^ and the VEGAN package (version 2.0-7).

## Results

### Wheat Yield Trends Under Long-Term N Application

**FIGURE 1:**
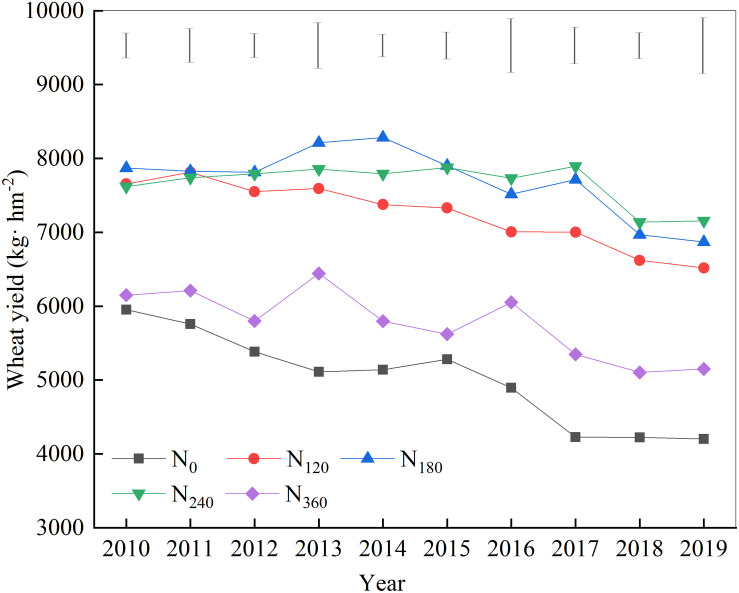
Effects of continuous application of different amounts of nitrogen fertilizer on wheat yield.

The yield of wheat grown on treatment plots with different N fertilizers showed a gradually decreasing trend over the 10 years of the study (Figure 1). During the first 6 years of continuous nitrogen application, wheat yields under the N_180_ treatment were invariably the highest, whereas from years 7 to 10, wheat yields under the N_240_ treatment were the highest. Throughout the treatment period, wheat yields under the N_120_ treatment tended to be lower than those in plots treated with N_180_ and N_240_.

### Responses of the Rhizosphere Soil Microbial Community to Long-Term N Application

**FIGURE 2:**
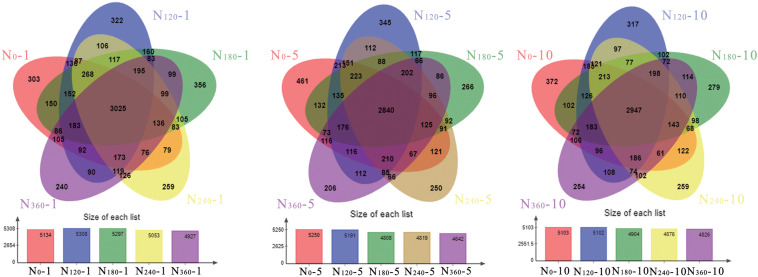
Rhizosphere soil microbial community responses to different long-term nitrogen fertilizer applications. *N_0_-1*, *N_0_-5*, and *N_0_-10* represent the first, fifth, and 10th year of the 0 kg N hm^–2^ treatment, respectively. A similar notation has been used for the other fertilizer treatments.

We found that N fertilizer had a significant effect on the number of bacterial species (Figure 2). During the first year of treatment, the number of bacterial OTUs under the N_120_ treatment was highest, whereas the N_180_-treated soil had the highest number of proprietary bacterial OTUs. Throughout the study period, we detected no significant changes in the number of bacterial species under the N_0_ treatments, whereas after 10 years, the number of OTUs in the soils treated with N_120_, N_180_, N_240_, and N_360_ had decreased by 3.84, 7.42, 3.50, and 2.05%, respectively. During the first year of N application, the numbers of proprietary bacterial OTUs in rhizosphere soils treated with N_0_ and N_180_ were 303 and 356, respectively. Following 5 and 10 years of continuous fertilization, the number of proprietary bacterial OTUs in rhizosphere soil treated with N_0_ had increased by 52.15 and 22.77%, respectively, whereas the number in soils treated with N_180_ had decreased by 25.28 and 21.63%, respectively.

Our determinations of the alpha richness and diversity of the rhizosphere communities at the phylum level are presented in Table 1. We detected no significant differences in the Chao richness indices of these communities in response to long-time N application, whereas the Shannon diversity indices had decreased significantly by the 10th year under the N_120_, N_180_, and N_240_ treatments.

**TABLE 1:** Alpha richness and diversity of the rhizosphere bacterial communities determined during long-term N application.

Index	Year	N_0_	N_120_	N_180_	N_240_	N_360_
Chao	1	34.94 ± 1.74a	32.88 ± 1.55a	36.13 ± 8.93a	32.75 ± 3.93a	31.88 ± 2.29a
	5	32.81 ± 1.57a	31.75 ± 0.96a	31.75 ± 1.32a	31.42 ± 0.79a	35.13 ± 7.98a
	10	35.77 ± 8.76a	32.63 ± 0.92a	31.96 ± 2.16a	30.77 ± 3.70a	30.65 ± 1.17a
Shannon	1	2.01 ± 0.04a	2.08 ± 0.03a	2.08 ± 0.02a	2.02 ± 2.02a	2.01 ± 0.09a
	5	2.03 ± 0.05a	2.06 ± 0.02a	2.04 ± 0.04a	2.08 ± 2.08a	2.01 ± 0.09a
	10	1.96 ± 0.06a	1.96 ± 0.04b	1.96 ± 0.03b	1.93 ± 1.93b	1.96 ± 0.03a

*Different lowercase letters after the same column value indicate that the difference between the various treatments under the same crop reached a significant level (P < 0.05). Chao reflects community richness and Shannon reflects community diversity. Five N treatments: N_0_ (0 kg N hm^–2^), N_120_ (120 kg N hm^–2^), N_180_ (180 kg N hm^–2^), N_240_ (240 kg N hm^–2^), and N_360_ (360 kg N hm^–2^).*

### Variations in the Proportion of Dominant Species Under Long-Time N Application

**FIGURE 3:**
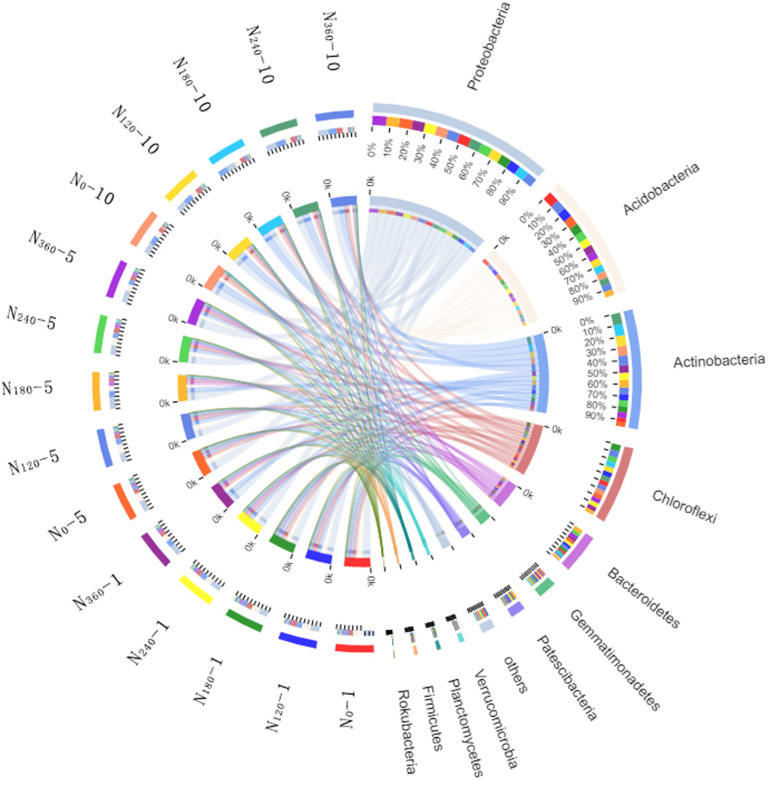
Proportions of the dominant bacterial phyla in different treatment samples. *N_0_-1*, *N_0_-5*, and *N_0_-10* represent the first, fifth, and 10th year of the 0 kg N hm^–2^ treatment, respectively. A similar notation has been used for the other fertilizer treatments. In the relation diagram between the Circos sample and species, the *small semicircle* (*left half circle*) represents the species composition in the sample. The *color of the outer ribbon* represents the group from which it comes. The *color of the inner ribbon* represents the species, and the *length* represents the relative richness of the species in the corresponding sample. The *large semicircle* (*right half circle*) indicates the distribution proportion of species in the different samples at the taxonomic level. The *color of the outer ribbon* represents the species, the *color of the inner ribbon* represents the different groups, and the *length* represents the distribution proportion of the sample in a certain species.

Variations in the trends of the average relative richness of the dominant bacterial species among the different treatments analyzed at the phylum level are shown in Figure 3. During the first year of application of the N_0_, N_120_, N_180_, N_240_, and N_360_ treatments, we found that the average relative richness values of *Acidobacteria* were 27, 24, 22, 19, and 18%, respectively. After 5 and 10 years of continuous localization, the average relative richness of *Acidobacteria* had gradually decreased with the increase in N fertilizer dosage, although there were no significant differences among the different treatments in the 10th year. The average relative richness of *Actinobacteria, Bacteroidetes*, and *Patescibacteria* initially showed a gradual increase with an increase in N fertilizer dosage, however, after 5 years of continuous N application, the richness had subsequently decreased with an increase in the N fertilizer dosage. The highest average relative richness of *Actinobacteria*, Bacteroidetes, and Patescibacteria was obtained under the N_180_ treatment.

**FIGURE 4:**
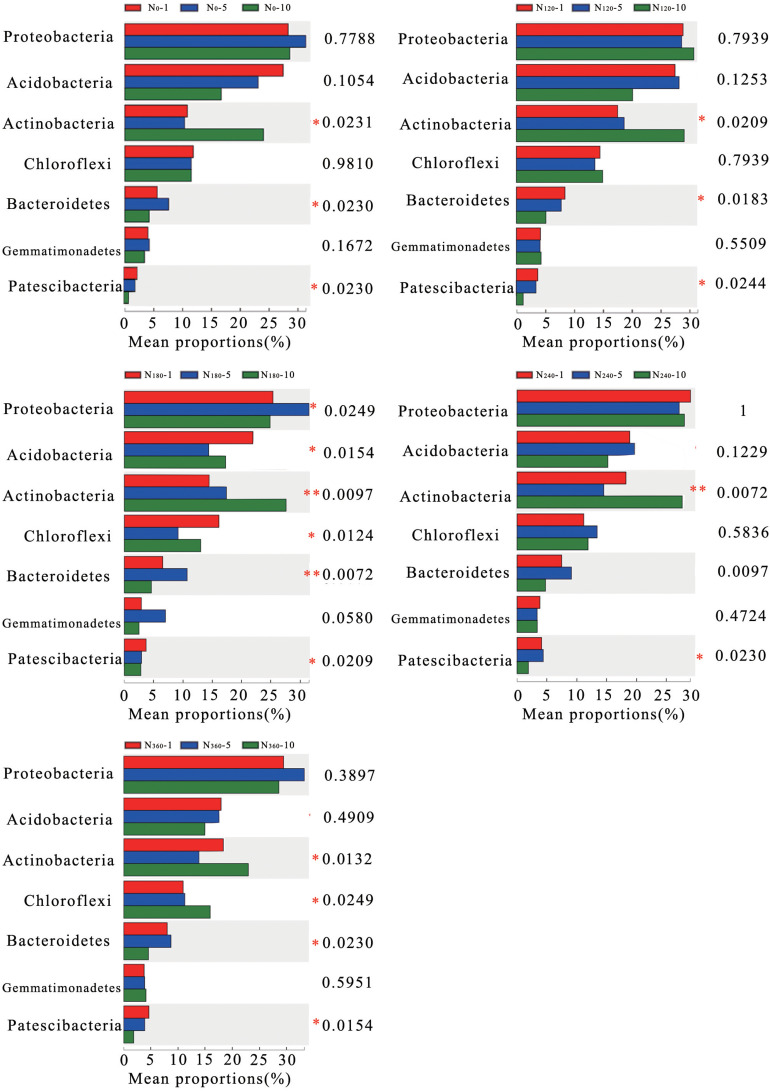
Correlations between the different long-term nitrogen applications and changes in the dominant phyla of the soil bacteria. *N_0_-1*, *N_0_-5*, and *N_0_-10* represent the first, fifth, and 10th year of the 0 kg N hm^–2^ treatment, respectively. A similar notation has been used for the other fertilizer treatments. *Y* represents the species names at the taxonomic level, *X* represents the average relative richness of the species in different groups, and the *columns of different colors* represent different groups. On the *far right* is the *P*-value: *0.01 < *P* ≤ 0.05; **0.001 < *P* ≤ 0.01; ***P ≤ 0.001.

The average relative richness of *Actinobacteria*, *Bacteroidetes, and Patescibacteria* gradually decreased with a prolongation of the treatment period (Figure 4), with those of *Bacteroidetes* and *Patescibacteria* decreasing significantly and the decreasing trend observed for *Bacteroidetes* reaching an extremely significant level under continuous treatment with N_180_. Similarly, in response to the different treatments, the average relative richness of *Actinobacteria* decreased significantly with a prolongation of N application and reached an extremely significant level under the N_180_ and N_240_ treatments.

### Effects of Environmental Characteristics on Rhizosphere Soil Bacterial Communities

**FIGURE 5:**
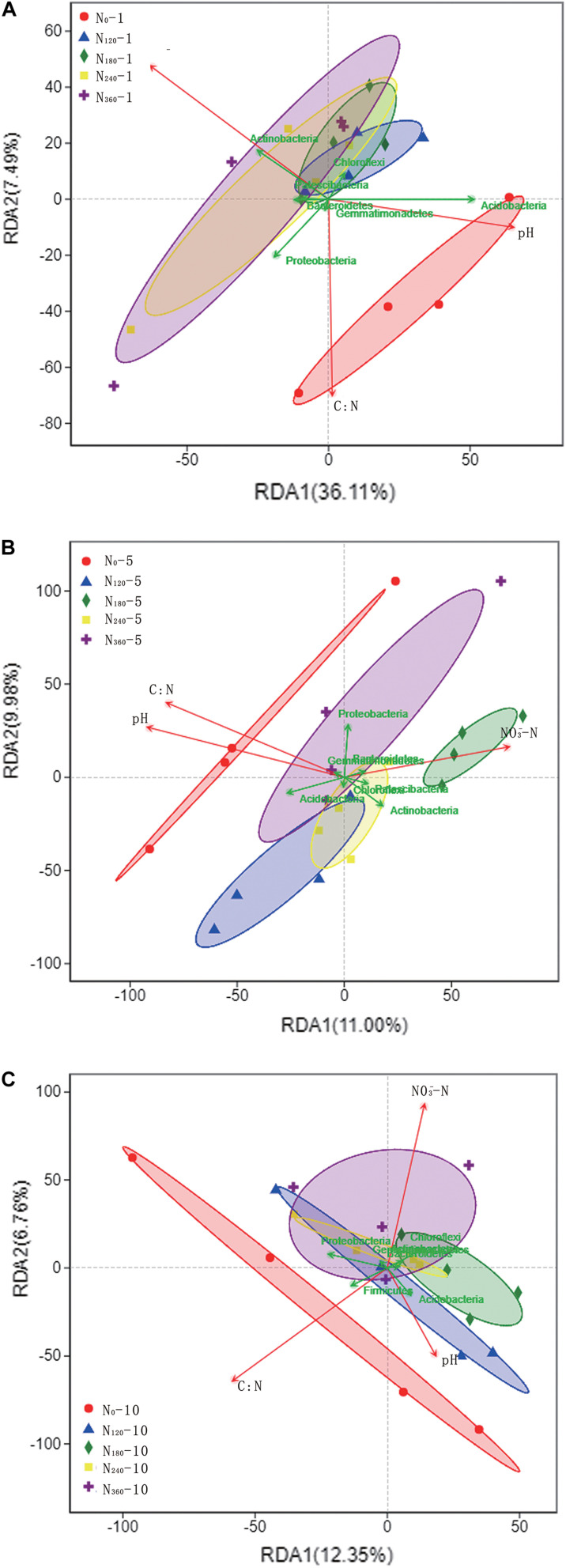
Correlations between dominant phyla of soil bacteria and environmental factors under different long-term nitrogen applications. *N*_*0*_-1, *N*_*0*_-5, and *N*_*0*_-10 represent the first, fifth and tenth year of the 0 kg N hm^−2^ treatment, respectively. A similar notation has been used for the other fertilizer treatments. Labels **(A–C)** represent the first, fifth and tenth year of fertilizer treatments respectively. The *points in the figure with different colors or shapes* represent the sample groups under different environments or conditions; The *red arrows* represent quantitative environmental factors, and the *length of the environmental factor arrows* can represent the degree of environmental factors’ influence on the species data (explanatory quantity). The included *angle between arrows* of environmental factors represents positive and negative correlation (acute angle: positive correlation; obtuse angle: negative correlation; right angle: no correlation); From the *sample point* to the *arrows* of the quantitative environmental factors, the distance between the projection point and the origin represents the relative influence of the environmental factors on the distribution of the sample community, and the *direction* of the *points* and *arrows* represents the positive and negative correlation.

Among the bacterial phyla, we found that *Actinobacteria*, *Proteobacteria*, *Acidobacteria*, and *Chloroflexi* showed a notably high relative richness. Soil NO_3_^–^, TN, and pH were used as the environmental factors of rhizosphere soil for 10 consecutive years (Table 2). During the first year of N application, we observed a gradual increase in the dispersion of species among the rhizosphere soil samples in response to increasing N applications, coinciding with the significant negative correlations between N input and the soil C/N ratio and pH and the significant positive correlations with soil NO_3_^–^ (Figure 5A). *Acidobacteria* showed a significantly positive correlation with soil pH, whereas *Actinobacteria* showed a significantly positive correlation with soil NO_3_^–^; Patescibacteria showed a positive correlation with soil NO_3_^–^.

**TABLE 2:** Effects of a continuous nitrogen fertilizer supply on rhizosphere soil characteristics.

Treatment	pH			C/N ratio (%)			NO_3_^–^ (mg/kg)		
	1st Year	5th Year	10th Year	1st Year	5th Year	10th Year	1st Year	5th Year	10th Year
N_0_	7.44a	7.40a	7.31a	5.59a	11.73a	14.08a	11.26d	12.51d	9.89e
N_120_	7.37a	7.25b	7.06b	5.16a	5.41b	6.84b	29.74c	26.99c	22.06d
N_180_	7.31a	7.23b	7.17ab	4.20bc	5.98b	5.95bc	32.58bc	30.64c	32.36c
N_240_	7.27a	7.21b	7.05b	4.06c	4.81b	6.01bc	34.59b	38.99b	40.08b
N_360_	7.34a	7.24b	7.00b	4.86ab	4.61b	4.54c	40.72a	51.79a	50.19a

*Different lowercase letters after the same column value indicate that the difference between the various treatments under the same crop reached a significant level (P < 0.05). Chao reflects community richness and Shannon reflects community diversity. Five N treatments: N_0_ (0 kg N hm^–2^), N_120_ (120 kg N hm^–2^), N_180_ (180 kg N hm^–2^), N_240_ (240 kg N hm^–2^), N_360_ (360 kg N hm^–2^).*

Subsequent to the initial five consecutive years of N localization, the difference in the dispersion of species between samples was lowest for soils treated with N_180_ and N_240_, whereas the dispersion of species between the samples treated with high N fertilization (N_360_) was higher. However, we were unable to detect significant correlations with any species within the dispersion interval of the N_360_ samples (Figure 5B). During this period, we found that *Actinobacteria*, *Bacteroidetes*, and *Patescibacteria* were positively correlated with soil NO_3_^–^, whereas Acidobacteria were positively correlated with soil pH and soil C/N ratio.

Following 10 consecutive years of N localization, we found that the dispersion of species between the samples was lowest and highest in the soils treated with N_240_ and N_360_, respectively, however, there were no significant correlations between the individual species within the dispersion interval of the N_360_ samples (Figure 5C). *Proteobacteria* showed a positive correlation with the soil C/N ratio, whereas *Chloroflexi* and *Actinobacteria* showed a significantly positive correlation with soil NO_3_^–^ and *Acidobacteria* showed a significantly positive correlation with soil pH.

Throughout the 10 consecutive years of N application under all N treatments, soil pH invariably showed a negative correlation with the soil NO_3_^–^ concentrations, and there was a consistent positive correlation between Acidobacteria and soil pH (Figure 5). Over the 10 years, we detected no significant effect of the N_0_ treatment on the rhizosphere soil microbial communities, whereas the species dispersion between the samples under long-term N_180_ and N_240_ treatments remained relatively low.

### Correlation Between Soil Environmental Factors and the Dominant Species of Rhizosphere Soil Bacteria

**FIGURE 6:**
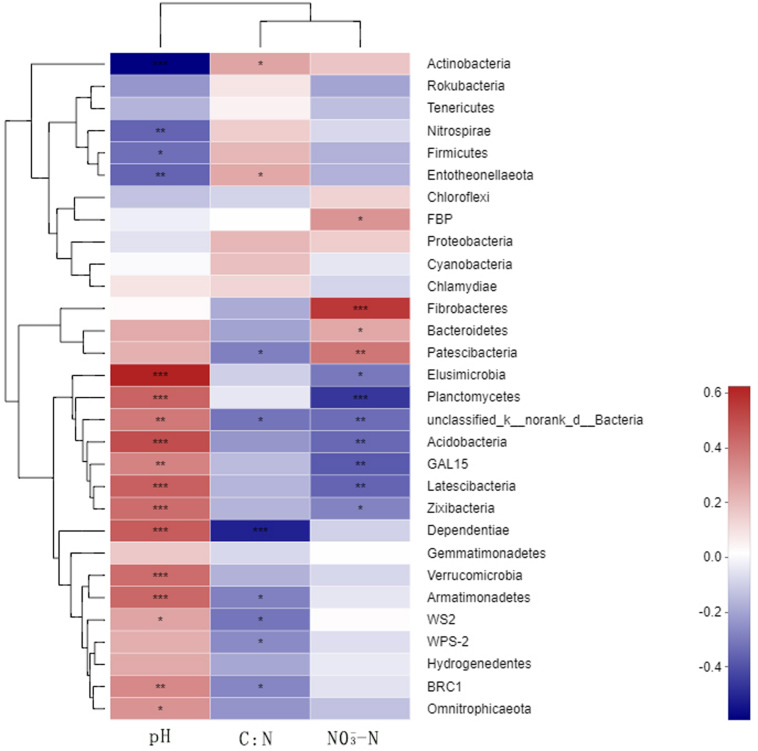
Relationships between the dominant soil bacteria and environmental factors. *N_0_-1*, *N_0_-5*, and *N_0_-10* represent the first, fifth, and 10th year of the 0 kg N hm^–2^ treatment, respectively. A similar notation has been used for the other fertilizer treatments. *X* and *Y* are the environmental factors and species, respectively, and the correlation *R*-value and *P*-value are obtained by calculation. The *R-*value is shown in *different colors* in the figure. If the *P*-value is < 0.05, it is marked with an *asterisk*. The *legend on the right* is the color interval of the different *R*-values. Cluster trees of the species and environmental factors are presented on the *left* and *upper parts*. *0.01 < *P* ≤ 0.05; **0.001 < *P* ≤ 0.01; ***P ≤ 0.001.

A correlation heat map generated to examine the relationships between the bacterial phyla and soil environmental factors revealed notable variations among the phyla (Figure 6). *Actinobacteria*, Nitrospirae, and Entotheonellaeota showed extremely significant negative correlations with soil pH, whereas Acidobacteria were significantly positively correlated with soil pH. Nitrospirae, Firmicutes, and Entotheonellaeota showed significant positive correlations and Patescibacteria showed an extremely significant negative correlation with the soil C/N ratio. Fibrobacteres and Patescibacteria showed extremely significant positive correlations with soil NO_3_^–^, whereas *Planctomycetes, unclassified_k_norank_d_Bacteria, Acidobacteria, GAL15*, and *Latescibacteria* showed extremely significant negative correlations. In contrast, we found *Proteobacteria* and *Gemmatimonadetes* to be unaffected by either the N fertilizer or the soil environmental factors.

### Correlations Among Yield, N Application, and Dominant Bacterial Species

**FIGURE 7:**
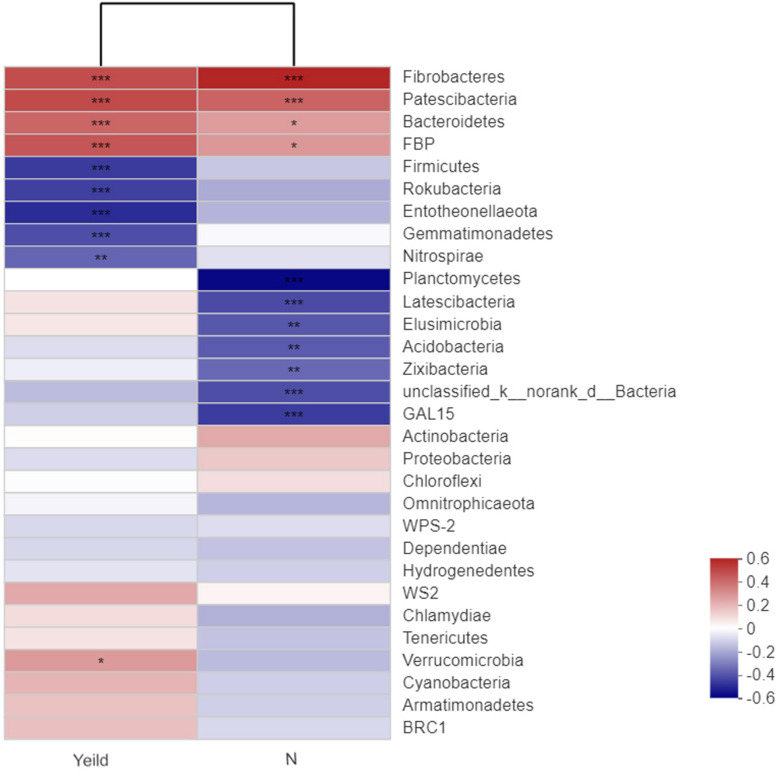
Correlations among the yield, nitrogen application, and dominant soil bacteria. *N_0_-1*, *N_0_-5*, and *N_0_-10* represent the first, fifth, and 10th year of the 0 kg N hm^–2^ treatment, respectively. A similar notation has been used for the other fertilizer treatments. The *R* value is shown in *different colors* in the figure. If the *P*-value is < 0.05, it is marked with an *asterisk*. The *legend on the right* is the color interval of the different *R*-values. Cluster trees of the species and environmental factors are presented on the *left* and *upper sides*. *0.01 < *P* ≤ 0.05; **0.001 < *P* ≤ 0.01; ***P ≤ 0.001.

Our analysis of the relationships between wheat yield and the rhizosphere soil bacterial species revealed that Fibrobacteres, Patescibacteria, Bacteroidetes, and FBP showed extremely significant positive correlations with yield (Figure 7). In addition, Fibrobacteres and Patescibacteria showed extremely significant positive correlations with N fertilizer treatment. Firmicutes, Rokubacteria, Entotheonellaeota, Gemmatimonadetes, and Nitrospirae showed extremely significant negative correlations with yield. The dominant bacterial species unclassified_k_norank_d_Bacteria, Acidobacteria, were significantly negatively correlated with N fertilizer treatment.

## Discussion

### Trends in Yield Under Different Levels of Long-Term N Application

Although N fertilization is a key factor in ensuring abundant crop yields, the widespread and excessive use of N fertilizers across China, particularly in northern China, has resulted in considerable negative effects. Long-term excessive N fertilizer inputs can reduce soil pH, facilitate soil erosion and hardening, and eventually lead to reductions in crop yields or even crop failures (Wang et al., 2017). Consistently, we found, in the present study, that with over 10 years of continuous fertilizer input, the wheat yields in plots receiving a high amount of N fertilizer (360 kg N hm^–2^) were invariably low. Indeed, the yields obtained with this level of fertilization did not differ significantly from those obtained with the N_0_ treatment, and the wheat yields obtained with both these treatments gradually decreased over time.

According to the established N fertilizer demands of wheat, a timely and an appropriate N fertilization are key factors in ensuring the maintenance of high wheat yields, and these factors also make an important contribution to the cultivation of the soil, which reduces costs and protects the environment (Reeves and Liebig, 2016). During the first 6 years of continuous N fertilizer application in the present study, we found that an N input of 180 kg hm^–2^ was conducive to achieving the highest crop yields. Starting from the seventh to the 10th year, the 240 kg hm^–2^ nitrogen input began to achieve the highest crop yield; this level of fertilization showed a close relationship with fertilizer volatilization and loss.

Previous studies have shown that the pathways of N fertilizer loss include nitrate leaching, nitrification/denitrification, and ammonia volatilization (Marchant et al., 2016). Among these processes, nitrate N leaching is the main form of soil N loss, which increases significantly in response to N applications of 100 and 200 kg hm^–2^, with the apparent quantity and rate of loss increasing significantly when the amount of N applied exceeds 150 kg hm^–2^. In northern China, this loss can be exacerbated by a higher frequency of drought, which also increases ammonia volatilization (Li et al., 2015). Thus, although a continuous N fertilizer input of 120 kg hm^–2^ can maintain the basic fertility of the soil, the crop often does not achieve the corresponding theoretical yields due to nutrient loss *via* absorption and transformation.

### Diversity and Composition of Rhizosphere Soil Bacterial Communities Under Different N Application Levels

Rhizosphere soil microorganisms are important catalysts for the transformation of soil nutrients, and their activity can effectively promote fertilizer decomposition and ammonium nitration to facilitate nutrient absorption by crop roots (Potthast et al., 2012). Although an appropriate input of N fertilizer can effectively promote bacterial richness and diversity (Yang et al., 2018), mismatches can develop between the degradation capacity of the rhizosphere soil bacteria and the amounts of material to be degraded as a consequence of the excessive application of the N fertilizer, thereby resulting in an excessive accumulation of fertilizer and rhizosphere soil hardening (Herzog et al., 2015). This, in turn, has the effect of inhibiting the activities of the soil bacteria and causing a rapid decline in the diversification of rhizosphere soil bacterial communities (De Carvalho et al., 2016). These effects were well demonstrated in the present study, in which we found that the number of bacterial OTUs in the rhizosphere soils treated with N_120_ and N_180_ increased to different degrees compared with the no N fertilizer input, whereas in response to an N fertilizer input of 360 kg hm^–2^, we observed a marked decrease in the number of bacterial OTUs.

*Acidobacteria* and *Proteobacteria* are the most prominent groups in rhizosphere soil bacterial communities and also the main rhizosphere soil bacteria associated with crop plants (Zhou et al., 2017). *Acidobacteria* are a group of oligotrophic bacteria that are typically found in nutrient-poor and highly acidic soil environments, and they have the capacity to degrade complex and recalcitrant carbon sources (Fierer et al., 2003). In the present study, we found that the species richness of *Acidobacteria* was highest in the rhizosphere soil under the N_0_ treatment, and this richness was significantly reduced following N fertilizer input and showed a further gradual trend of decrease in response to increasing N fertilizer input.

The N fertilizer is rapidly solubilized in the rhizosphere soil to form NO_3_^–^, which is readily absorbed by the crop root systems, and in response to the spike in soil NO_3_^–^, the rhizosphere bacteria must mount a short-term reaction to deal with the initial stress induced by fertilization, the magnitude of which increases with an increase in N fertilizer input (Fujii, 2014; Ellen et al., 2009). Among the other prominent bacterial phyla, we found that the average relative richness of *Patescibacteria*, *Bacteroidetes*, and *Actinobacteria* showed a trend of gradual increase with increased N fertilizer inputs during the first year of the present study, and *Patescibacteria* and *Actinobacteria* showed significant positive correlations with soil NO_3_^–^. On the basis of these observations, it can thus be inferred that short-term N fertilizer input affects the predominant rhizosphere soil bacterial species by altering the content of NO_3_^–^ in soil.

### Response Mechanism of Rhizosphere Soil Bacteria Under Continuous Supply of N Fertilizer at Different Dosages

A wide variety of compounds released by plant roots can result in changes in the organic compounds in the rhizosphere soil and create unique microenvironments for soil microorganisms, which in turn affects the rhizosphere soil bacteria activity (Jia et al., 2015). It is well understood that soil biological activity plays an important role in soil fertility in unique microenvironments (Xu et al., 2009). Previous studies have shown that the total soluble sugars, free amino acids, soluble phenolic acids, and organic acids in the rhizosphere soil are mainly derived from the root exudates of wheat and microbial metabolism (Walker et al., 2003). Thus, the formation of wheat roots depends on the decomposition and transformation of nutrients by the rhizosphere soil microorganisms.

It has been established that long-term continuous input of the N fertilizer results in decreases in the diversity and size of the rhizosphere soil microbial communities (Zhang et al., 2016). During the 10 years of continuous treatment, the number of OTUs in the populations of rhizosphere soil bacteria gradually decreased in response to higher rates of N fertilization, and whereas the populations of the dominant bacterial species involved in N fertilizer decomposition were promoted, the overall richness of the dominant species declined (Zhou et al., 2015). With the exception of the N_0_ and N_360_ treatments, the diversity of the rhizosphere soil bacterial community was significantly reduced in response to the continuous application of the N fertilizer (Table 1).

Continuous N fertilizer input alters the nutrient status of the soil, and this status can serve as an index to indicate how environmental factors influence the rhizosphere soil microbial community (Luo et al., 2017). Studies have shown that the soil pH, organic C, N availability, and other soil properties have various effects on bacterial diversity (Horn et al., 2014). One of the consequences of the long-term application of N fertilizer is a decrease in soil pH, which is presumed to lead to a decrease in diversity, based on the fact that the soil pH is positively correlated with bacterial diversity in arable soils and that most bacterial taxa exhibit relatively narrow growth tolerances, particularly within the pH 4–7 range (Johannes et al., 2010).

In the present study, we found that Proteobacteria, *Actinobacteria*, Acidobacteria, Chloroflexi, and Bacteroidetes were negatively correlated with the soil pH to varying degrees. Acidobacteria, some species of which are fastidious oligotrophic bacteria, are known to be suppressed following N input (Kielak et al., 2008). In this study, we found that the average relative richness of *Actinobacteria* increased significantly with an increase in N fertilizer dosage and an increase the year of N application, and *Actinobacteria* were extremely significantly correlated with the soil pH. Thus, trends in the average relative richness of *Actinobacteria* can be considered indicative of the fact that the long-term application of N leads to a decrease in soil pH.

Rhizosphere soil bacteria have various mechanisms that are deployed in response to the presence of N and phosphate–potassium fertilizers, and the inputs of these fertilizers can have both favorable and unfavorable effects on different bacterial communities (Rousk et al., 2010). In the present study, we found that the average relative richness of the dominant bacteria, along with that of *Actinobacteria*, in the rhizosphere soil of wheat decreased significantly under the long-term application of N. Previous research has shown that the richness of bacteria such as Patescibacteria and Bacteroidetes has a positive effect on N-containing biosynthetic products (Lorig-Roach et al., 2017), and we found that the richness of bacteria in these two phyla showed a very significant correlation with wheat yield in the first 5 years of continuous N application, during which time the average relative richness of *Patescibacteria* and *Bacteroidetes* initially increased and then decreased with an increase in N application, reaching the highest value under N_180_. Thus, a decrease in the relative richness of the dominant species of rhizosphere soil bacteria is probably one of the factors leading to a decrease in wheat yield under long-term N fertilization. However, a limitation of this study is that it was conducted at only a single site in the North China Plain. Thus, we were unable to make multipoint comparisons, which would have enabled us to assess the influence of factors such as soil type and climate.

## Conclusion

The numbers of soil bacteria OTUs decreased to varying extents in response to the long-term application of different levels of N fertilizer, and wheat yields were significantly correlated with the average relative richness of *Patescibacteria* and *Bacteroidetes*. An appropriate N fertilizer input (N_180_) could still maintain a reasonably high average relative richness. Short-term N fertilizer input was found to affect the predominant bacterial species *via* altering the soil NO_3_^–^ contents. Long-term N fertilization can significantly affect most of the dominant soil bacterial species *via* changing the soil pH. The richness of *Actinobacteria* can serve as an indicator of a decreased soil pH caused by long-term N fertilization. We also found that bacteria in the phyla Proteobacteria and Gemmatimonadetes appear to be unaffected by the N fertilizer and the soil environmental factors we assessed (pH, C/N ratio, and NO_3_^–^).

## Data Availability Statement

The datasets generated for this study can be found in the NCBI, PRJNA613763.

## Author Contributions

All authors listed have made a substantial, direct and intellectual contribution to the work, and approved it for publication.

## Conflict of Interest

The authors declare that the research was conducted in the absence of any commercial or financial relationships that could be construed as a potential conflict of interest.

## Footnotes

1 http://drive5.com/uparse/

2 http://rdp.cme.msu.edu/

3 http://circos.ca/

4 http://www.R-project.org

## References

[B1] Ai C. Liang G. Q. Sun J. Wang X. B. He P. Zhou W. (2013). Different roles of rhizosphere effect and long-term fertilization in the activity and community structure of ammonia oxidizers in a calcareous fluvo-aquic soil. *Soil Biol. Biochem.* 57 30–42. 10.1016/j.soilbio.2012.08.003

[B2] Bremner J. M. Mulvaney C. S. (1982). “Nitrogen—total,” in *Methods of Soil Analysis. Part 2. Chemical and Microbiological Properties, American Society of Agronomy*, ed. Page A. L. (Madison, WI: Soil Science Society of America), 595–624.

[B3] Caporaso J. G. Kuczynski J. Stombaugh J. Bittinger K. Bushman F. D. Costello E. K. (2010). QIIME allows analysis of high-throughput community sequencing data correspondence QIIME allows analysis of high-throughput community sequencing data/Intensity normalization improves color calling in SOLiD sequencing. *Nat. Methods Nat. Publ. Gr.* 7 335–336. 10.1038/nmeth0510-335 PMC3156573 20383131

[B4] Chen J. W. Li J. Yan J. J. Li H. X. Zhou X. (2014). Abundance and community composition of Ammonia-Oxidizing bacteria and archaea under different regeneration scenarios in chinese loess plateau. *Soil Sci.* 179 369–375. 10.1097/SS.0000000000000080

[B5] Chen S. Waghmode T. R. Sun R. Kuramae E. E. Hu C. (2019). Root-associated microbiomes of wheat under the combined effect of plant development and nitrogen fertilization. *Microbiome* 7 136–148. 10.1186/s40168-019-0750-2 31640813 PMC6806522

[B6] Cui Z. L. Zhang F. S. Chen X. P. Miao Y. X. Li J. Shi L. (2008). On-farm evaluation of an in-season nitrogen management strategy based on soil N min test. *Field Crops Res.* 105 48–55. 10.1016/j.fcr.2007.07.008

[B7] De Carvalho T. S. Da Concei O. Jesus E. Barlow J. Gardner T. A. Soares I. C. (2016). Land use intensification in the humid tropics increased both alpha and beta diversity of soil bacteria. *Ecology* 97 2760–2771. 10.1002/ecy.1513 27859123

[B8] DeAngelis K. M. Brodie E. L. DeSantis T. Z. Andersen G. L. Lindow S. E. Firestone M. K. (2009). Selective progressive response of soil microbial community to wild oat roots. *Isme J.* 3 168–178. 10.1038/ismej.2008.103 19005498

[B9] Ellen K. Thomas B. Esther E. Dörr N. Georg G. Norbert L. (2009). Response of total and nitrate-dissimilating bacteria to reduced n deposition in a spruce forest soil profile. *Fems Microbiol. Ecol.* 67 444–454. 10.1111/j.1574-6941.2008.00632.x 19220860

[B10] Eo J. Park K. (2016). Long-term effects of imbalanced fertilization on the composition and diversity of soil bacterial community. *Agric. Ecosyst. Environ.* 231 176–182. 10.1016/j.agee.2016.06.039

[B11] Fierer N. Schimel J. P. Holden P. A. (2003). Variations in microbial community composition through two soil depth profiles. *Soil Biol. Biochem.* 35 167–176. 10.1016/S0038-0717(02)00251-1

[B12] Fujii K. (2014). Soil acidification and adaptations of plants and microorganisms in Bornean tropical forests. *Ecol. Res.* 29 371–381. 10.1007/s11284-014-1144-3

[B13] Gasparatos D. Roussos P. A. Christofilopoulou E. Haidouti C. (2011). Comparative effects of organic and conventional apple orchard management on soil chemical properties and plant mineral content under mediterranean climate conditions. *J. Soil Sci. Plant Nutr.* 11 105–117. 10.4067/s0718-95162011000400008 27315006

[B14] Ge Y. Zhang J. B. Zhang L. M. Yang M. He J. Z. (2008). Long-term fertilization regimes affect bacterial community structure and diversity of an agricultural soil in northern China. *J. Soils Sedi.* 8 43–50. 10.1065/jss2008.01.270

[B15] Geisseler D. Scow K. M. (2014). Long-term effects of mineral fertilizers on soil microorganisms-a review. *Soil Biol. Biochem.* 75 54–63. 10.1016/j.soilbio.2014.03.023

[B16] Haichar F. Z. Marol C. Berge O. Rangel-Castro J. I. Prosser J. I. Balesdent J. (2008). Plant host habitat and root exudates shape soil bacterial community structure[J]. *Isme J.* 2 1221–1230. 10.1038/ismej.2008.80 18754043

[B17] Han J. P. Shi J. C. Zeng L. Z. Xu J. M. Wu L. S. (2017). Impacts of continuous excessive fertilization on soil potential nitrification activity and nitrifying microbial community dynamics in greenhouse system. *J. Soils Sedi.* 17 471–480. 10.1007/s11368-016-1525-z

[B18] Herzog S. Wemheuer F. Wemheuer B. Daniel R. (2015). Effects of fertilization and sampling time on composition and diversity of entire and active bacterial communities in german grassland soils. *PLoS One* 10:e145575. 10.1371/journal.pone.0145575 26694644 PMC4687936

[B19] Horn D. J. V. Okie J. G. Buelow H. N. Gooseff M. N. Takacs-Vesbach C. D. (2014). Soil microbial responses to increased moisture and organic resources along a salinity gradient in a polar desert. *Appl. Environ. Microbiol.* 80 3034–3043. 10.1016/j.fcr.2017.03.002 24610850 PMC4018898

[B20] Hu Y. T. Hao M. D. Wei X. R. Chen X. Zhao J. (2016). Contribution of fertilisation, precipitation, and variety to grain yield in winter wheat on the semiarid Loess Plateau of China. *Acta Agric. Scand. Sect. B Soil Plant Sci.* 66 406–416. 10.1080/09064710.2016.1149215

[B21] Islam M. R. Hossain M. B. Siddique A. B. Rahman M. T. Malika M. (2015). Contribution of green manure incorporation in combination with nitrogen fertilizer in rice production. *SAARC J. Agric.* 12 134–142. 10.3329/sja.v12i2.21925

[B22] Jansson J. K. Hofmockel K. S. (2019). Soil microbiomes and climate change. *Nat. Rev. Microbiol.* 18 35–46. 10.1038/s41579-019-0265-7 31586158

[B23] Jason G. Murray W. John D. (2005). Computational improvements reveal great bacterial diversity and high metal toxicity in soil. *Science* 309 1387–1390. 10.1126/science.1112665 16123304

[B24] Jia X. Zhao Y. H. Wang W. K. He Y. (2015). Elevated temperature altered photosynthetic products in wheat seedlings and organic compounds and biological activity in rhizopshere soil under cadmium stress. *Sci. Rep.* 5:14426. 10.1038/srep14426 26395070 PMC4585777

[B25] Kielak A. Pijl A. S. Veen J. A. V. Kowalchuk G. A. (2008). Phylogenetic diversity of Acidobacteria in a former agricultural soil. *Isme J.* 3 378–382. 10.1038/ismej.2008.113 19020558

[B26] Koranda M. Kaiser C. Fuchslueger L. Kitzler B. Sessitsch A. Zechmeister-Boltenstern S. (2013). Seasonal variation in functional properties of microbial communities in beech forest soil. *Soil Biol. Biochem.* 60 95–104. 10.1016/j.soilbio.2013.01.025 23645937 PMC3618437

[B27] Lagomarsino A. Knapp B. A. Moscatelli M. C. De Angelis P. Grego S. Insam H. (2007). Structural and functional diversity of soil microbes is affected by elevated [CO2] and N addition in a poplar plantation. *J. Soils Sedi.* 7 399–405. 10.1065/jss2007.04.223

[B28] Lennon J. T. Jones S. E. (2011). Microbial seed banks: the ecological and evolutionary implications of dormancy. *Nat. Rev. Microbiol.* 9 119–130. 10.1038/nrmicro2504 21233850

[B29] Li Q. Yang A. Wang Z. Roelcke M. Chen X. Zhang F. (2015). Effect of a new urease inhibitor on ammonia volatilization and nitrogen utilization in wheat in north and northwest China. *Field Crops Res.* 175 96–105. 10.1016/j.fcr.2015.02.005

[B30] Lorig-Roach N. Still P. C. Coppage D. Compton J. E. Crews P. (2017). Evaluating nitrogen-containing biosynthetic products produced by saltwater culturing of several california littoral zone gram-negative bacteria. *J. Nat. Prod.* 80 2304–2310. 10.1021/acs.jnatprod.7b00302 28777571 PMC5687060

[B31] Luo D. Liu S. Shi Z. M. Feng Q. H. He J. S. (2017). Soil microbial community structure in Picea asperata plantations with different ages in subalpine of western Sichuan, Southwest China. *Ying Yong Sheng Tai Xue Bao* 28 519–527. 10.13287/j.1001-9332.201702.028 29749160

[B32] Luo S. Yu L. Yu L. Ying Z. Wang J. (2016). Effects of reduced nitrogen input on productivity and N2O emissions in a sugarcane/soybean intercropping system. *Eur. J. Agron.* 81 78–85. 10.1016/j.eja.2016.09.002

[B33] Ma L. Zhang W. F. Ma W. Q. Velthof G. L. Oenema O. Zhang F. S. (2013). An analysis of developments and challenges in nutrient management in china. *J. Environ. Qual.* 42 951–961. 10.2134/jeq2012.0459 24216347

[B34] Marchant H. K. Holtappels M. Lavik G. Ahmerkamp S. Winter C. Kuypers M. M. (2016). Coupled nitrification–denitrification leads to extensive N loss in subtidal permeable sediments. *Limnol. Oceanogr.* 61 1033–1048. 10.1002/lno.10271

[B35] Maron J. L. Marler M. Klironomos J. N. Cleveland C. C. (2011). Soil fungal pathogens and the relationship between plant diversity and productivity. *Ecol. Lett.* 14 36–41. 10.1111/j.1461-0248.2010.01547.x 21073641

[B36] Mchugh T. A. Schwartz E. (2015). Changes in plant community composition and reduced precipitation have limited effects on the structure of soil bacterial and fungal communities present in a semiarid grassland. *Plant Soil* 388 175–186. 10.1007/s11104-014-2269-4

[B37] Mori H. Maruyama F. Kato H. Toyoda A. Dozono A. Ohtsubo Y. (2014). Design and experimental application of a novel non-degenerate universal primer set that amplifies prokaryotic 16S rRNA genes with a low possibility to amplify eukaryotic rRNA genes. *DNA Res.* 21 217–227. 10.1093/dnares/dst052 24277737 PMC3989492

[B38] Nan X. Tan G. Wang H. Gai X. (2016). Effect of biochar additions to soil on nitrogen leaching, microbial biomass and bacterial community structure. *Eur. J. Soil Biol.* 74 1–8. 10.1016/j.ejsobi.2016.02.004

[B39] National bureau of statistics (2019). *China Statistical Yearbook.* Beijing: National bureau of statistics.

[B40] Oburger E. Gruber B. Wanek W. Watzinger A. Stanetty C. Schindlegger Y. (2016). Microbial decomposition of 13C- labeled phytosiderophores in the rhizosphere of wheat: mineralization dynamics and key microbial groups involved. *Soil Biol. Biochem.* 98 196–207. 10.1016/j.soilbio.2016.04.014

[B41] Ortiz-Castro R. Contreras-Cornejo H. A. Macias-Rodriguez L. Lopez-Bucio J. (2009). The role of microbial signals in plant growth and development. *Plant Signal. Behav.* 4 701–712. 10.4161/psb.4.8.9047 19820333 PMC2801380

[B42] Page A. L. Miller R. H. Keeney Dennis R. (1982). Methods of soil analysis. *Catena* 15 99–100. 10.1007/BF02869702

[B43] Potthast K. Hamer U. Makeschin F. (2012). In an Ecuadorian pasture soil the growth of *Setaria sphacelata*, but not of soil microorganisms, is co-limited by N and P. *Appl. Soil Ecol.* 62 103–114. 10.1016/j.apsoil.2012.08.003

[B44] Reeves J. L. Liebig M. A. (2016). Soil pH and exchangeable cation responses to tillage and fertilizer in dryland cropping systems. *Commun. Soil Sci. Plant Anal.* 47 2396–2404. 10.1080/00103624.2016.1243706

[B45] Rousk J. Bååth E. Brookes P. C. Lauber C. L. Lozupone C. (2010). Soil bacterial and fungal communities across a pH gradient in an arable soil. *ISME J.* 4 1340–1351. 10.1038/ismej.2010.58 20445636

[B46] Shaw K. (1959). Determination of organic carbon in soil and plant material. *Eur. J. Soil Sci.* 10 316–326. 10.1111/j.1365-2389.1959.tb02353.x

[B48] Sun M. Xiao T. Ning Z. Xiao E. Sun W. (2015). Microbial community analysis in rice paddy soils irrigated by acid mine drainage contaminated water. *Appl. Microbiol. Biotechnol.* 99 2911–2922. 10.1007/s00253-014-6194-5 25408313

[B49] Tesoriero A. J. Duff J. H. Wolock D. M. Spahr N. E. Almendinger J. E. (2009). Identifying pathways and processes affecting nitrate and orthophosphate inputs to streams in agricultural watersheds. *J. Environ. Qual.* 38 1892–1900. 10.2134/jeq2008.0484 19643755

[B50] Walker T. S. Bais H. P. Grotewold E. Vivanco J. M. (2003). Root exudation and rhizosphere biology. *Plant Physiol.* 132 44–51. 10.1104/pp.102.019661 12746510 PMC1540314

[B51] Wang H. Y. Zhang Y. T. Chen A. Liu H. Zhai L. Lei B. (2017). An optimal regional nitrogen application threshold for wheat in the North China Plain considering yield and environmental effects. *Field Crops Res.* 207 52–61. 10.1016/j.fcr.2017.03.002

[B52] Wei G. Xuebin Q. Yatao X. Ping L. Mathias A. Yan Z. (2018). Effects of reclaimed water irrigation on microbial diversity and composition of soil with reducing nitrogen fertilization. *Water* 10 365–381. 10.3390/w10040365

[B53] Xu Y. Wang G. Jin J. Liu J. Zhang Q. Liu X. (2009). Bacterial communities in soybean rhizosphere in response to soil type, soybean genotype, and their growth stage. *Soil Biol. Biochem.* 41 919–925. 10.1016/j.soilbio.2008.10.027

[B54] Yang Y. Dou Y. An S. (2018). Testing association between soil bacterial diversity and soil carbon storage on the Loess Plateau. *Sci. Total Environ.* 626 48–58. 10.1016/j.scitotenv.2018.01.081 29335174

[B55] Zhang B. Gao Q. S. Xu S. Q. Ma L. Tian C. J. (2016). Long-term effect of residue return and fertilization on microbial biomass and community composition of a clay loam soil. *J. Agric. Sci.* 154 1051–1061. 10.1017/S0021859615001008

[B56] Zhou J. Guan D. W. Zhou B. K. Zhao B. S. Ma M. C. Qin J. (2015). Influence of 34-years of fertilization on bacterial communities in an intensively cultivated black soil in northeast China. *Soil Biol. Biochem.* 90 42–51. 10.1016/j.soilbio.2015.07.005

[B57] Zhou Y. Li J. Ross Friedman C. Wang H. (2017). Variation of soil bacterial communities in a chronosequence of rubber tree (Hevea brasiliensis) plantations. *Front. Plant Sci.* 8:849–861. 10.3389/fpls.2017.00849 28611794 PMC5447074

